# Biomechanical Stability of Third-Generation Adjustable Suture Loop Devices Versus Continuous Loop Button Device for Cortical Fixation of ACL Tendon Grafts

**DOI:** 10.1177/23259671241240375

**Published:** 2024-04-03

**Authors:** Adrian Deichsel, Lara Leibrandt, Michael J. Raschke, Matthias Klimek, Simon Oeckenpöhler, Elmar Herbst, Christoph Kittl, Johannes Glasbrenner

**Affiliations:** * Department of Trauma, Hand and Reconstructive Surgery, University Hospital Münster, Münster, Germany; Investigation performed at University Hospital Münster, Münster, Germany

**Keywords:** ACL reconstruction, adjustable loop button, continuous loop button, cortical fixation

## Abstract

**Background:**

Concerns regarding the primary stability of early adjustable loop button (ALB) devices for cortical fixation of tendon grafts in anterior cruciate ligament reconstruction (ACLR) have led to the development of new implant designs.

**Purpose:**

To evaluate biomechanical stability of recent ALB implants in comparison with a continuous loop button (CLB) device.

**Study Design:**

Controlled laboratory study.

**Methods:**

ACLR was performed in a porcine model (n = 40) using 2-strand porcine flexor tendons with a diameter of 8 mm. Three ALB devices (Infinity Button [ALB1 group]; Tightrope II RT [ALB2 group]; A-TACK [ALB3 group]) and 1 CLB device (FlippTack with polyethylene suture) were used for cortical tendon graft fixation. Cyclic loading (1000 cycles up to 250 N) with complete unloading were applied to the free end of the tendon graft using a uniaxial testing machine, followed by load to failure. Elongation, stiffness, yield load, and ultimate failure load were recorded and compared between the groups using a Kruskal-Wallis test with post hoc Dunn correction.

**Results:**

Elongation after 1000 cycles at 250 N was similar between groups (ALB1, 4.5 ± 0.7 mm; ALB2, 4.8 ± 0.8 mm; ALB3, 4.5 ± 0.6 mm; CLB, 4.5 ± 0.8 mm), as was load to failure (ALB1, 838 ± 109 N; ALB2, 930 ± 89 N; ALB3, 809 ± 103 N; CLB, 842 ± 80 N). Stiffness was significantly higher in the ALB1 group compared with the CLB group (262.3 ± 21.6 vs 229.3 ± 15.1 N/mm; *P* < .05). No significant difference was found between the 4 groups regarding yield load. Constructs failed either by rupture of the loop, breakage of the button, or rupture of the tendon.

**Conclusion:**

The tested third-generation ALB devices for cortical fixation in ACLR withstood cyclic loading with complete unloading without significant differences to a CLB device.

**Clinical Relevance:**

The third-generation ALB devices tested in the present study provided biomechanical stability comparable with that of a CLB device. Furthermore, ultimate failure loads of all tested implants exceeded the loads expected to occur in the postoperative period after ACLR.

Cortical suspensory fixation using a suture button has become a fixation technique used frequently in anterior cruciate ligament (ACL) reconstruction (ACLR).^15,43,44^ The first generation of suspensory devices was equipped with a continuous suture loop (continuous loop button [CLB]). Later, adjustable loop button (ALB) devices were introduced, promising an increased tendon-to-bone interface, especially in short femoral tunnels, as well as a faster application with reduced operation time.

The mechanism of the adjustable suture loop varies depending on the manufacturer of the implant: the Chinese finger trap (CFT) mechanism consists of a suture loop inside an outer helically braided suture that tightens when tension is applied, locking the inner suture in place.^28^ Locked suture loop (LSL) devices rely on compression of the suture loop between the button and the suture loop itself or compression of the loop in an opening in the button.

In contrast to the advantage of simple and minimally invasive application,^2,4,19,26^ unfavorable primary stability of ALB devices using the CFT mechanism with increased elongation under cyclic loading has been reported.^3,6,42^ More precisely, biomechanical studies have indicated that CFT loops depend on continuous tension and loosen under cyclic loading and unloading, as is assumed to occur to the tendon graft during the postoperative period after ACLR.^22^

Recently, changes in implant designs have been made by different manufacturers of ALB, with new suture loop designs promising improved primary stability. Therefore, the purpose of the present study was to evaluate the primary stability of contemporary ALB devices in ACLR and to compare them with graft fixation with a CLB device. It was hypothesized that third-generation ALB devices would provide favorable biomechanical stability compared with a CLB device.

## Methods

### Graft Fixation

**Figure 1.**
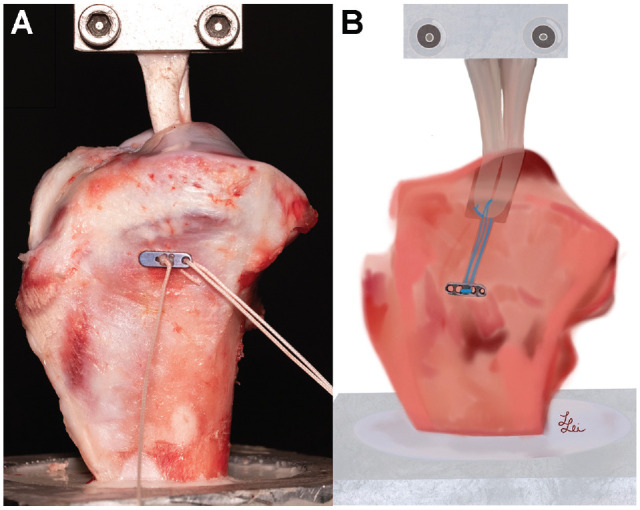
Cortical fixation of (A) a soft tissue tendon graft in dual diameter bone tunnel of 40 mm total length with (B) a tendon-to-bone interface of 20 mm length, with the free end of the tendon graft fixed to a material testing machine by a cryoclamp.

Ethics approval was waived for this study. ACLR was performed in a porcine knee model (n = 40) as previously established.^22^ Porcine knee and lower leg specimens were obtained from a local butcher, who confirmed adequate health and comparable age of all specimens used. Porcine knee specimens were gently thawed at 7°C for 24 hours. After removal of the surrounding soft tissues, the proximal tibiae were embedded into an aluminum mount using synthetic resin (RenCast FC 52/53 A ISO and RenCast FC 53 B Polyol; Gößl and Pfaff). Superficial porcine flexor tendons were trimmed to a double loop with a diameter of 8 mm and a length of 100 mm. The diameter of the tendon was measured using a standardized sizing device (±0.5 mm; Karl Storz), and the free ends of the tendon graft were tied over a length of 20 mm (Figure 1).

A transosseous K-wire was inserted at the anatomic insertion site of the ACL to create a drill hole of 40 mm length with an angulation of 50° in relation to the plane of the knee joint. Canulated drill bits were used to create a dual diameter socket, with the inner diameter corresponding to the manufacturer’s instructions of each tested implant. The proximal 20 mm of the tunnel were drilled to an outer diameter of 8 mm. The button-tendon construct was inserted into the tunnel and tensioned according to the manufacturer’s instructions. Envelope randomization was used to determine the order of testing.

### Implants

**Figure 2.**
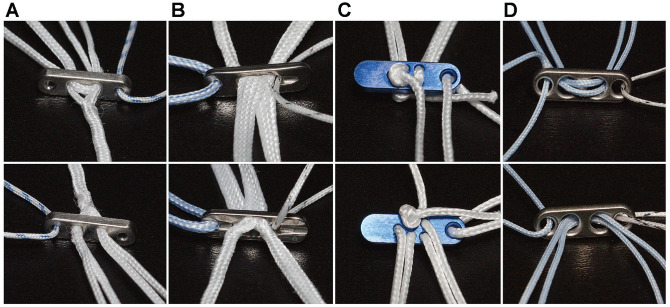
Details of cortical fixation devices from the front (top row) and back (bottom row): (A) Infinity Button; (B) Tightrope II RT; (C) A-TACK; and (D) custom-made continuous-loop button using a FlippTack with a double-looped and hand-knotted polyethylene suture (No. 2 FiberWire).

Three ALB devices and 1 CLB device were used for cortical fixation of ACL tendon grafts. The CLB construct corresponded to a fixation technique used in our clinical routine and consisted of a titanium suture button (FlippTack; Karl Storz) with a double-looped and hand-knotted polyethylene suture (No. 2 FiberWire; Arthrex).^37^ All implants were purchased commercially from the manufacturers. Buttons varied in size and thickness, with lengths from 11 to 13 mm, widths from 3 to 4 mm, and thickness from 1.5 to 1.6 mm. Details of the 4 implant groups evaluated in the present study are as follows (Figure 2):

- ALB1 group: Infinity Button (CONMED);
- ALB2 group: Tightrope II RT (Arthrex);
- ALB3 group: A-TACK (Karl Storz);
- CLB group: FlippTack with polyethylene suture (No. 2 FiberWire).

The ALB1 and ALB2 implants used a CFT mechanism for locking the length of the loop, while the ALB3 implant used an LSL mechanism.

### Biomechanical Testing

An electric uniaxial testing machine (model Z005; Zwick/Roell) with a 10-kN sensor (load cell accuracy, ±0.03%) was used for biomechanical testing. The mount containing the embedded porcine tibia was fixed to the base of the machine with 2 clamps. The proximal end of the graft was fixed to the testing machine using a cryoclamp 20 mm above the joint line (Figure 1). Cyclic loading was performed at a speed of 100 mm/s. Preconditioning was performed with 10 cycles at 250 N. Cyclic loading was performed with 1000 cycles between 0 and 250 N.^18^ Subsequently, load to failure was applied at a speed of 25 mm/min. Elongation and load were recorded continuously during all tests. Stiffness was determined by the slope of the linear portion of the load-displacement curve during load to failure. Yield load was defined as the point on the force-displacement curve at which elastic deformation ended and plastic deformation of the tendon/construct occurred (between 4% and 6% of elongation according to Józsa^25^ and Martin et al^29^). Mode of failure was documented macroscopically.

### Statistical Analysis

An a priori power analysis was performed using G*Power Version 3.1.^17^ To detect a difference of 100 N between group means at a standard deviation of 60 N, a sample size of 10 would lead to a power of at least 90%. The assumed standard deviations were based on previously reported studies on graft fixation strategies in porcine knee models.^16^

Extraction of biomechanical parameters from test data was performed using Matlab (Version R2020a; MathWorks). Statistical analysis was performed using Prism (Version 8; GraphPad software). Results are presented as mean and standard deviation. Distribution of the data was assessed using histograms and the Shapiro-Wilk test. Nonnormally distributed groups were compared using the Kruskal-Wallis test with post hoc Dunn correction. The significance level was set at *P* < .05.

## Results

**Figure 3.**
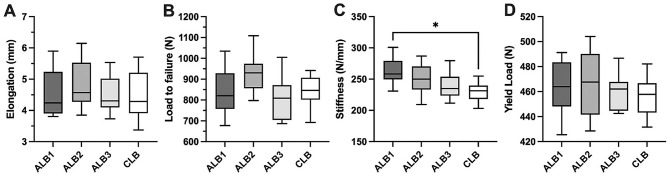
Results of biomechanical testing for (A) elongation after cyclic loading at 250 N, (B) load to failure, (C) stiffness, and (D) yield load. Boxplots present the mean (horizontal line), standard deviation (top and bottom of the box), and range (whiskers). *Statistically significant difference between groups (*P* < .05). ALB, adjustable loop button; CLB, continuous loop button.

Results of the biomechanical testing are summarized in Figure 3 and Table 1. After 1000 cycles of loading between 0 and 250 N, elongation was 4.5 ± 0.7 mm in the ALB1 group, 4.8 ± 0.8 mm in the ALB2 group, 4.5 ± 0.6 mm in the ALB3 group, and 4.5 ± 0.8 mm in the CLB group. Load to failure was 838 ± 109 N in the ALB1 group, 930 ± 89 N in the ALB2 group, 809 ± 103 N in the ALB3 group, and 842 ± 80 N in the CLB group. No significant differences were observed between the groups for elongation during cyclic loading or ultimate failure load. Stiffness was 262.3 ± 21.6 N/mm in the ALB1 group, 250.3 ± 24.7 N/mm in the ALB2 group, 239.6 ± 21.7 N/mm in the ALB3 group, and 229.3 ± 15.1 N/mm in the CLB group. A statistically significant difference was found between the ALB1 and the CLB groups (*P* < .05). Yield load was 465 ± 21 N in the ALB1 group, 466 ± 27 N in the ALB2 group, 460 ± 15 N in the ALB3 group, and 455 ± 16 N in the CLB group, with no significant group differences.

**Table 1:** Elongation, Load to Failure, Stiffness, and Yield Load According to Device^*a*^

Group	Elongation, mm	Load to Failure, N	Stiffness, N/mm	Yield Load, N
ALB1	4.5 ± 0.7	838 ± 109	262.3 ± 21.6	465 ± 21
ALB2	4.8 ± 0.8	930 ± 89	250.3 ± 24.7	466 ± 27
ALB3	4.5 ± 0.6	809 ± 103	239.6 ± 21.7	460 ± 15
CLB	4.5 ± 0.8	842 ± 80	229.3 ± 15.1	455 ± 16

a Data are presented as mean ± SD. ALB, adjustable loop button; CLB, continuous loop button.

Construct failure occurred by rupture of the tendon graft or the suture loop or by dislocation or breakage of the cortical button. The predominant failure mode in each group was dislocation of the button into the bone tunnel (n = 8/10) in the ALB1 group, breakage of the button (n = 7/10) in the ALB2 group, rupture of the adjustable loop (n = 5/10) in the ALB3 group, and rupture of the continuous loop (n = 7/10) in the CLB group. However, there was no significant difference regarding ultimate failure load between all groups.

Furthermore, no damage to the specimens was observed other than the aforementioned failure modes. Explicitly, there was no slippage of the tendon at the cryoclamp and no damage to the cortex of the bone during cyclic loading.

## Discussion

The most important finding of the present study was that third-generation ALB devices for cortical fixation of ACL tendon grafts demonstrated favorable primary stability with no significant difference in comparison with a continuous loop device.

Primary stability of ALB button devices has been assessed in a variety of biomechanical studies with different study protocols.^20,36,41^ First, isolated testing of the implant led to lower elongation and higher load to failure in comparison to in vitro ACLR.^6,12,19,24,36^ However, the influence of the interface between the implant and the cortical bone or the tendon graft is not considered in isolated implant testing. Second, in numerous biomechanical studies, cyclic loading was performed without complete unloading.^4,19,23,30^ A previous study on forces acting in the knee joint has shown that, during gait, there is periodic complete unloading of the ACL.^32^ Furthermore, rehabilitation protocols after ACLR are typically composed of partial weightbearing as well as passive mobilization during the early postoperative phase, indicating probable unloading of the ACL.

In a biomechanical investigation by Glasbrenner et al^22^ that included cyclic loading with complete unloading in a porcine knee model, a significantly increased elongation of earlier ALB devices, utilizing the CFT mechanism, was found in comparison to a CLB (same result as in the present study). The elongation for CFT devices in that study was 8.1 and 6.1 mm, respectively, both significantly greater than for an LSL device with 4.7 mm and a CLB with 4.1 mm.^22^ The testing protocols during cyclic testing varied between the study by Glasbrenner et al^22^ (stepwise increasing load) and the present study (continuous load), limiting the comparison of absolute values. However, in the present study, elongation of ALB utilizing the CFT mechanism (groups ALB1 and ALB2) ranged from 4.5 to 4.8 mm and was not significantly different than an LSL device (ALB3, 4.5 mm) and the CLB device (4.5 mm), indicating that current changes in CFT designs led to improved primary stability of these implants.

In a further biomechanical investigation, 2 ALB devices were compared in a porcine model of femoral ACL graft fixation, with an elongation of 3.42 ± 1.34 and 3.39 ± 0.92 mm, respectively.^19^ However, the authors performed extensive preloading (150 N for 5 minutes) and did not perform unloading of the constructs (1000 cycles from 50 to 250 N). Furthermore, load to failure was determined by testing the isolated implant (862 ± 64 N and 1879 ± 126 N, respectively). In comparison, the highest ultimate failure load in the present study was 930 ± 89 N (seen in the ALB2 group), indicating only a limited influence of the tendon-to-implant and implant-to-bone interfaces on the failure loads. However, the forces acting on the ACL during gait have been determined to range between 0 and 454 N.^31,34,40^ In the present study, all of the tested implants exceeded these values, both regarding load to failure as well as yield load, indicating sufficient maximum failure loads of all tested implants. A significant difference in stiffness was observed between the ALB1 and CLB groups. This could be due to differences in suture material, button design, or locking mechanism (CFT vs knotted loop). However, the clinical relevance of this finding is questionable, especially since no significant differences were found regarding load to failure and yield load between the different groups. Furthermore, implants displayed different failure mechanisms under load to failure. We hypothesize this to be attributable to the design of the buttons, which varied in size and thickness, with lengths from 11 to 13 mm, widths from 3 to 4 mm, and thickness from 1.5 to 1.6 mm.

Recent clinical studies have investigated the difference between ALB and CLB regarding clinical outcome after ACLR. A prospective randomized controlled trial of 43 patients found no significant differences in functional scores (International Knee Documentation Committee and Lysholm), as well as clinical stability tests.^5^ Furthermore, retrospective studies have found noninferiority of ALB graft fixation in comparison with CLB.^13,27,39^ A systematic review and meta-analysis concluded that the scarce evidence suggests no difference in knee stability and subjective knee function between ALB and CLB.^14^

In previous generations of ALB, different approaches were presented to prevent slippage of the constructs and to improve primary stability. Retensioning of the ALB, as well as tying a knot over the button, after reaching the desired loop length has been shown to significantly reduce elongation.^8,9,33^ Considering the results of the present study and current literature, additional measures such as tying a knot over the button do not appear to be necessary when using the implants tested in the present study.

### Limitations

Several limitations should be considered when interpreting the results of the present study. Previous studies have shown that the porcine knee anatomy is sufficiently similar to that of the human knee^38^ and that porcine flexor tendons possess similar biomechanical properties to human hamstring tendons,^11^ making the porcine knee model a setup used frequently to assess biomechanics of orthopaedic implants in knee surgery.^10,18,19,21^ A graft diameter of 8 mm was chosen for this study, resembling the typical thickness of a hamstring ACL graft. Considering that some implants failed by damage to the implant-to-bone interface and that bone density in the porcine model is significantly higher in comparison with human specimens,^1^ this may have biased the load to failure toward higher values.^35^ In the present study, elongation was calculated by tracking the displacement of the testing machine crossbar over time. A recent study showed that, in such a test setup, the elongation was significantly higher than the actual graft slippage as measured by optical tracking at the tunnel aperture.^7^ However, both values were highly correlated in that study and can therefore be used to compare elongation between groups in the present study. Nevertheless, values from this study cannot be transferred directly to the clinical setting. Finally, comparison between implants was limited to the timepoint of implantation. Possible influences of the different materials on tendon-bone healing cannot be deduced.

## Conclusion

Third-generation adjustable suture loop devices for cortical fixation of ACL tendon grafts withstood cyclic loading with complete unloading in the present biomechanical study, indicating favorable primary stability. Furthermore, ultimate failure loads of all tested implants exceeded the loads expected to occur in the postoperative period after ACLR.
